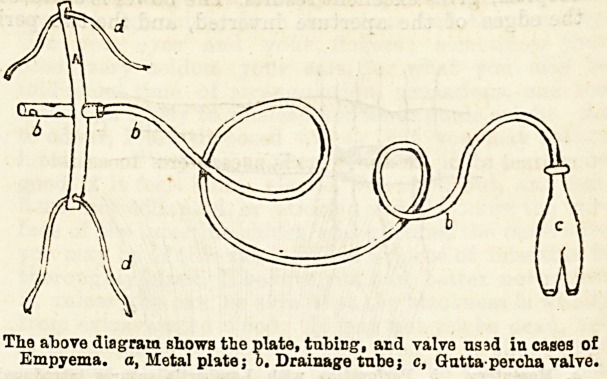# Treatment of Pleurisy

**Published:** 1893-03-11

**Authors:** 


### THE ROYAL SOUTHERN HOSPITAL, LIVERPOOL.

### Treatment of Pleurisy.

In discussing the treatment of pleurisy we feel that we are standing upon debatable ground, and whether we are to confine ourselves to the purely physician's side of the question, or to that of the surgeon, or perhaps a happy combination of both, the latter we feel will be the more acceptable.

How often we see those slight attacks of pleurisy, even perhaps with a certain amount of effusion, get quite well under purely medicinal treatment; other cases, again, where do as we will the fluid (effusion) will not become absorbed, and it is in these cases that the surgeon must endeavour to effect a cure. So that in this discussion we shall first of all treat of those cases where no surgical interference is necessary, and then go on to discuss those cases of effusion and suppuration (empyæma) which need surgical interference.

First, then, with reference to those cases where we have the typical signs of pleurisy with or without a certain amount of effusion; we, of course, do not refer to those very mild cases where you get simply a pain in the side, with slight friction sound, which will be at once relieved by the application of a poultice, and the patient feel nothing more of the trouble. We do not see these cases in hospital, unless with some complication. We refer more particularly to those cases where we have an acute inflammation of the pleura, with exudation of lymph and effusion of serum into the chest cavity, with dulness on percussing, perhaps extending up to the fifth costal cartilage, showing that we have a certain amount of effusion; there is not a great deal. We have no displacement of the heart and no dyspnœa in such a case as this. We endeavour to get absorption of the fluid by medicinal means; there is no urgency about the case, so that surgical interference is not immediately called for. Tonics in the shape of quinine and iron are generally prescribed, and counter-irritation to the affected side, either painting with Tr. iodi or the application of blistering fluid, or even the old-fashioned poultice, which is not to be despised; it affords great comfort if nothing else. We can call to mind one case particularly which impressed us very favourably with the efficacy of diuretics, a method of treatment so often suggested in text-books. The case was one with a considerable amount of effusion, dulness extending up to the third costal cartilage, and the treatment in this case was as follows: Potassii iodidi gr. 40, potassii acetatis ʒiij., inf. gent. ad. ℥viij.; one ounce of this mixture to be taken three times a day, and the following application for the chest to be applied warm daily, viz.: Tr. iodi ʒiv., spr. rect. ℥ij. The result was certainly very favourable, the fluid became absorbed in about a fortnight. The diet usually consists of milk and milk puddings, with tea and bread and butter, and as they improve fish and meat is allowed. Stimulants are not given, unless there is some indication for their use. When convalescent they are sent away into the country to recruit. This treatment generally suffices in these mild cases.

We come now to those cases with a large amount of effusion, and in discussing these an interesting point presents itself, viz., what should be the indication for surgical interference? Should we persevere *ad infinitum* with medicinal treatment, and after having exhausted all our resources in this direction, tap the chest, or should we wait for respiratory embarrassment, or should the presence of a large amount of effusion alone, without any complication, be sufficient to warrant one in aspirating? We generally follow the latter, and do not persevere with drugs or wait for respiratory embarrassment.

In these days of antiseptics the dangers of the slight operation are almost nil.

The operation is carried out as follows: A medium-sized hollow needle is taken, and the aspirator—(and here we will say just a few words with reference to it). We generally use what we believe is called "Maw's," that is, with an indiarubber cork, which can be fitted to any bottle. We find that it is much more convenient than "Dieulafoy's Aspirator," which consists of a large syringe with two stopcocks—the aspirator, then, is fitted up, viz., the indiarubber cork is put into the bottle, the air is exhausted from the bottle by means of the air-pump, the stopcock is then turned off, thus completely sealing the bottle, and making it absolutely air-tight; an indiarubber tube is then attached by one end to the metal tube on the cork, and by the other end to the hollow needle before mentioned. The patient is prepared as follows: The point for aspiration is selected, either between the fifth and sixth ribs in the mid-axillary line, or just below the angle of the scapula. This part is thoroughly washed with an antiseptic—preferably carbolic lotion (strength 1 in 20)—and then frozen to diminish the pain caused by the puncture of the needle. The freezing is accomplished either by holding a piece of ice to the side or by the ether spray. Either of these means will deaden the pain. The needle is then taken between the forefinger and thumb of right hand, and plunged quickly into side between the ribs, in the position above mentioned, keeping as near as possible to the centre of the intercostal space, thereby avoiding the artery. The stopcock is turned, and the serous fluid immediately runs into the vacuum chamber, viz., the bottle. The fluid is allowed to run until the pleural cavity is empty, or until the patient complains of pain or shows a tendency to cough, indicating that the lung is expanding, and impinging against the needle. The needle is at once drawn out. Before drawing out, however, a little salicylic wool is got ready, and some collodion poured over it, and immediately the needle leaves the puncture in the skin the collodion and salicylic wool is placed over it to prevent any chance of air reaching the pleural cavity. A piece of lint just sufficient to cover the wool is placed next. This is fastened to the chest wall by means of strapping, and over all is put a flannel binder or bandage. The patient is then allowed to lie down and rest. This operation really causes very little discomfort to the patient; the pain is absolutely diminished by the freezing. Should, however, any faintness come on during the operation, one ounce of brandy is given; and, invariably, after it is all over, a stimulant is given in the form either of brandy or whisky. This is all the surgical treatment that is generally required. Aspiration may be called for again should a reaccumulation of fluid take place. The medicinal treatment consists generally in giving tonics such as quinine and iron, or any of the bitters. In discussing this aspiration, a point worth mentioning is the disappearance of the fluid by removing a few drops. We occasionally find that by the insertion of a needle of a hypodermic syringe, and drawing off a few drops of the fluid, more for diagnostic purposes than for any curative effect it may have, that we get an absorption of the remaining fluid, due, we presume, to the relief of pressure on the serous membrane. Last, but not least by any means, we come to suppuration (empyæma) in the pleural cavity. This, perhaps, is one of the most unsatisfactory conditions that the surgeon is called upon to treat. Who cannot call to mind cases of empyæma that have gone on discharging stinking foul pus for weary months together, until at last lardaceous disease carries off the little sufferer, or perhaps recovery takes place, but with a collapsed lung and contracted, deformed chest, the lung bound down to the side of the vertebral column by adhesions, preventing apposition of the pleural surfaces, and thus a large cavity is left to secrete pus. This is opened, and a drainage tube inserted, and the cavity allowed to drain into antiseptic dressings, which keeps the discharge aseptic at first, but which invariably gets septic in a few days; then the vigorous washing out to try and improve matters, but more often than not ending in complete failure. This was a common picture, or perhaps is even now. Happily we, at the above hospital, can tell a very different story, certainly in the majority of cases; occasionally a case will resist all attempts at drainage, and resection of ribs has to be resorted to in the end, but these cases are certainly rare.

The method we adopt is as follows, and is, if we are not mistaken, peculiar to the hospital, as it owes its origin to one of the physicians:—

The patient is anæsthetised either with chloroform or ether; the point selected for the incision is between the fifth and sixth ribs in the mid-axillary line; the part is thoroughly cleansed with antiseptic lotions; a small incision is then made through the skin and superficial structures; a good-sized trochar is taken, and thrust into the pleural cavity; the point of the trochar is pulled out, the canula being left *in situ*, the pus spurting out. A probe is now passed through the canula into the cavity of the pleural, the canula now withdrawn, and the probe left *in situ*; the probe thus leaves a guide for a pair of dressing forceps, which are pushed into the opening made by the trochar, and the handles opened out after the method suggested by Hilton for enlarging wounds. This is necessary for the insertion of the drainage tube, which can be done quite easily by this method, but not otherwise, for it is not so easy as it looks. The tube is inserted, and about two or three inches is passed into the pleural cavity. The drainage tube is not of the ordinary kind used for wounds, but a little thicker, in fact, a little better quality. It will require to be of some length to reach from the patient's chest into a bottle under the bed. Probably about five feet would be long enough. This tube is passed through a metal cap about $1\frac{1}{2}$ inches square, and the indiarubber tubing fits the hole in the cap very tightly to prevent the tube slipping. The cap is of pliable metal, so that it can be moulded accurately to the chest wall. It has a slot at each end (or top and bottom) for the passage of tapes, to fix it to chest wall, and thus fix the drainage tube also, and prevent it from slipping out of the pleural cavity. The other end of the tube is passed into a bottle containing a little antiseptic fluid, and the pus is allowed to drain away for the first twelve hours, after which time a small gutta-percha valve is attached to the end of

The above diagram shows the plate, tubing, and valve used in cases of Empyema. *a*, Metal plate; *b*, Drainage tube; *c*, Gutta-percha valve.

the tube in the bottle. This valve will allow the pus to escape, but will also form a vacuum in the pleural cavity, and thus tend to cause and help expansion of the lung. This is not disturbed in any way, except for any complication that may arise. Should the pus get fœtid the pleural cavity is washed out. This is very easily accomplished. All one has to do is to remove the valve from the end of the tube, and, after having emptied any pus or discharge from the bottle, fill it up with any antiseptic lotion (we generally use lot. boracis, warm), and then elevate the bottle. The tube being absolutely air-tight, when it goes into pleural cavity we have a siphon action, and the lotion runs into pleural cavity. We have only to lower the bottle again to get a return of the fluid. As the discharge diminishes, the part of the tube in the pleural cavity is gradually shortened, until it can be dispensed with altogether. The part is then dressed with a little ung. boracis on lint and salicylic wool until the wound is healed. The patient is prescribed some tonic (any of those mentioned), and sent away to a convalescent home to recruit. We find that the large majority of the cases so treated have complete expansion of the lung, and no contraction of the affected side of the chest. On measurement there may possibly be a difference of a quarter or half an inch, but this is of no consequence. We, of course, occasionally have to resort to resection of ribs, but this is a formidable surgical operation, and deserves an article to itself.

## Figures and Tables

**Figure f1:**